# Fluorofenidone Inhibits UUO/IRI-Induced Renal Fibrosis by Reducing Mitochondrial Damage

**DOI:** 10.1155/2022/2453617

**Published:** 2022-03-20

**Authors:** Xiaohua Liao, Xin Lv, Yan Zhang, Yuanyuan Han, Jiajia Li, Jianhua Zeng, Damu Tang, Jie Meng, Xiangning Yuan, Zhangzhe Peng, Lijian Tao, Yanyun Xie

**Affiliations:** ^1^Department of Nephrology, Xiangya Hospital, Central South University, Changsha Hunan, China; ^2^Affiliated Drum Tower Hospital of Nanjing University, Nanjing Jiangsu, China; ^3^Hunan Key Lab of Organ Fibrosis, Changsha Hunan, China; ^4^Hamilton Urologic Oncology Research Center (HUORC), St. Joseph's Hospital and Department of Medicine, McMaster University, Hamilton, Ontario, Canada; ^5^Department of Respirology, Third Xiangya Hospital, Central South University, Changsha Hunan, China; ^6^National International Collaborative Research Center for Medical Metabolomics, Xiangya Hospital, Central South University, Changsha Hunan, China

## Abstract

**Objective:**

Mitochondrial damage contributes to extracellular matrix (ECM) deposition and renal fibrosis. In this study, we aimed (1) to investigate whether fluorofenidone (AKF-PD) can attenuate mitochondrial damage in two renal fibrosis models: unilateral ureteral obstruction (UUO) and renal ischemia-reperfusion injury (IRI), and (2) to explore the underlying mechanism.

**Method:**

Mitochondrial damage and renal lesions were analyzed in the UUO and IRI models. Mitochondrial energy metabolism, mitochondrial biogenesis, and oxidative stress were measured to assess the effect of AKF-PD on mitochondrial damage and to explore the underlying mechanism. In addition, HK-2 cells were stimulated with TGF-*β* with and without AKF-PD. The mitochondrial morphology, mtROS, ATP contents, and redox-related proteins were then examined.

**Results:**

In both UUO and IRI models, AKF-PD relieved renal fibrosis, maintained mitochondrial structure, and increased mitochondrial DNA copy numbers. The protection was associated with (1) sustaining mitochondrial energy metabolism, evident by elevations of tricarboxylic acid (TCA) cycle enzymes and mitochondrial respiratory chain complexes; (2) improving mitochondrial biogenesis with increases of TFAM, NRF1, PGC-1*α*, and SIRT1; and (3) reducing mitochondrial oxidative stress likely via regulating SOD2, SIRT3, and NOX4 expressions. In HK-2 cells treated with TGF-*β*, AKF-PD protected mitochondria along with improving mitochondrial morphology, enhancing ATP production, reducing mtROS, and regulating SOD2, SIRT3, and NOX4 expression.

**Conclusion:**

We demonstrate that AKF-PD inhibited renal fibrosis at least in part via protecting mitochondria from damages developed in the UUO and IRI models. The mitochondrial protection was associated with sustaining mitochondrial energy metabolism, improving mitochondrial biogenesis, and reducing mitochondrial oxidative stress. This research verified the protective effect of AKF-PD on mitochondria in the UUO and IRI models and elaborated the underlying mechanism.

## 1. Introduction

Renal fibrosis is caused by excessive deposition of extracellular matrix (ECM), which contributes to the progression of chronic kidney disease (CKD) [1]. Globally, CKD accounts for more than 10% of morbidity and the number of CKD patients is increasing [2]. Management of renal fibrosis remains challenging with few effective prevention and treatment measures available. Therefore, it is important to develop effective drugs against renal fibrosis to prevent the progression of CKD.

Clinically, renal fibrosis can be resulted from complex mechanisms related to a variety of kidney injuries, such as urinary obstruction and ischemia [3, 4]; both disease courses are commonly modeled by unilateral ureteral obstruction (UUO) and ischemia-reperfusion injury (IRI) procedures [5]. The mechanisms leading to renal fibrosis in both animal models (UUO and IRI) are likely complex; accumulative evidence reveals mitochondrial damage as a pathological cause [6, 7]. In the UUO and IRI models, renal fibrosis is progressed along with mitochondrial damage, evident by disrupted energy metabolism, impaired mitochondrial biogenesis, and oxidative stress [8, 9].

The major function of mitochondria is to produce ATP via fatty acid oxidation (FAO), tricarboxylic acid (TCA) cycle, and mitochondrial respiratory chain [10]. However, the impact of these energy metabolic pathways on renal fibrosis induced by UUO and IRI remains incompletely understood. It has been suggested that improvement of mitochondrial biogenesis and inhibition of oxidative stress should be considered to reduce mitochondrial damage and sustain ATP production [11]. This strategy is relevant in attenuation of renal fibrosis; it is thus appealing to investigate approaches and small molecular compounds possessing such activities.

Fluorofenidone (AKF-PD), a pyridone and an antifibrotic agent, is undergoing a phase II clinical trial for the treatment of liver fibrosis in China. AKF-PD shows therapeutic effects in various experimental models of fibrosis in the liver, kidney, and lung [12–14]. The compound possesses a variety of pharmacological activities, such as antioxidative, antiapoptotic, and anti-inflammatory activities [15]. Consistent with AKF-PD's broad antifibrotic actions and the important contributions of mitochondrial dysfunction to renal fibrosis, our recent evidence demonstrated that AKF-PD can reduce mitochondrial damage by inhibiting mitochondrial oxidative stress in the folic acid-induced renal fibrosis model, where downregulation of NADPH oxidase 4 (NOX4) expression may play a role [16]. In view of renal fibrosis being conveyed by a variety of diseases [17], the observed protection of mitochondria from damage by AKF-PD in renal fibrosis caused by folic acid [16] calls for investigations on AKF-PD-derived mitochondrial protection in renal fibrosis under other disease conditions. Urinary obstruction and IRI are important clinical causes of renal fibrosis; a role of AKF-PD in protecting renal fibrosis in both conditions via protecting mitochondria remained unclear.

By using mouse UUO and IRI models for fibrosis as well as human renal tubular epithelial (HK-2 cells), we have systemically investigated AKF-PD's antifibrotic actions via sustaining mitochondrial morphology and function and further explored the underlying mechanisms. We reported here that AKF-PD inhibited renal fibrosis caused by UUO and IRI at least in part via reducing mitochondrial damage, evident by AKF-PD ability in maintaining mitochondrial morphology, sustaining energy metabolism, improving mitochondrial biogenesis, and keeping oxidative stress under control.

## 2. Materials and Methods

### 2.1. Materials

AKF-PD (lot No. 20190810) was purchased from Hai**k**ou Pharma (Hai**k**ou, China). ATP assays were obtained from Beyotime Biotechnology (Shanghai, China). Recombinant human transforming growth factor-*β* (TGF-*β*) was provided by PeproTech (Rocky Hill, USA). MitoSOX Deep Red was purchased from Invitrogen (New York, USA). The following antibodies for immunohistochemistry and western blotting were used: **s**irtuin 1 (SIRT1), NOX4, E-cadherin, superoxide dismutase 2 (SOD2), and **s**irtuin 3 (SIRT3) antibodies, which were purchased from Proteintech (San Diego, USA). Other antibodies were from Abcam (Cambridge, UK): collagen I, collagen III, fibronectin (FN), total OXPHOS complexes, and 4-hydroxynonenal (4HNE). Anti-alpha smooth muscle actin (*α*-SMA) antibody and anti-GAPDH antibody were obtained from Sigma (St. Louis, MO, USA) and Cell Signaling Technology (Boston, MA, USA), respectively. All other chemicals were of analytical grade.

### 2.2. Animals and Treatment

Male C57BL/6 mice were purchased from the Silaike Laboratory (Shanghai, China). The animal studies were carried out in the Medical Genetics Laboratory of Central South University with approval by the Animal Care and Use Committee of Central South University.

We have previously demonstrated that AKF-PD had no effect on sham-operated animals [18]. In this research, healthy 6–8-week-old male mice were divided randomly into three groups: sham, UUO operation, and UUO operation with AKF-PD treatment. For surgery, the mice were anesthetized with 2–4% isoflurane administered through inhalation. Mice were subjected to left UUO or sham surgery as previously described [18]. One day later, the UUO+AKF-PD group was gavaged AKF-PD (500 mg/kg/day, 0.5% CMC-Na as the solvent). The sham group and the UUO group were administered vehicle (0.5% CMC-Na). Mice were euthanized with a high concentration of CO_2_ through inhalation on day 14 after UUO surgery, and the kidney tissues were harvested for pathologic examination, immunohistochemistry, and other tests.

To construct the IRI model, healthy 6–8-week-old male mice were divided randomly into three groups: control, IRI operation, and IRI operation with AKF-PD treatment. For surgery, the mice were anesthetized with 2–4% isoflurane administered through inhalation. Both renal pedicles were exposed under flank incisions; then, both kidneys were clamped for 30 mins, during which body temperature was maintained at 36.5°C–37.5°C using a temperature-controlled heating device. One day after IRI operation, the IRI+AKF-PD group was gavaged AKF-PD (500 mg/kg/day, 0.5% CMC-Na as the solvent). The control group and the IRI group were administered vehicle (0.5% CMC-Na) simultaneously. Mice were euthanized with a high concentration of CO_2_ through inhalation on day 7 after IRI surgery. Serum was harvested for renal function testing, and the kidney tissues were used for pathologic examination, immunohistochemistry, and other tests.

### 2.3. Cell Culture and Treatments

HK-2 cells were purchased from American Type Culture Collection (ATCC, USA). Cells were cultured in F12 from HyClone (Logan, Utah, USA) supplemented with 10% FBS, 100 U/mL penicillin, and 100 *μ*g/mL streptomycin from Life Technologies (Carlsbad, California, USA) at 37°C in a humidified atmosphere of 5% CO_2_ and 95% air. The cells were seeded on 6-well culture plates and were separated randomly into 3 groups: normal group, TGF-*β* group, and AKF-PD group. After pretreatment with or without AKF-PD (400 *μ*g/mL) for 24 h, HK-2 cells were incubated with or without TGF-*β* (10 ng/mL) for 48 h to induce mitochondrial damage.

### 2.4. Histopathology

As reported in our previous study [19], mouse kidney tissue was embedded in paraffin to prepare sections for hematoxylin and eosin (HE) and Masson trichrome staining. Scoring on HE slides and Masson slides was used to evaluate the degree of tubulointerstitial injury and tubulointerstitial fibrosis, respectively.

### 2.5. Immunohistochemistry

The slides were incubated with primary antibodies against collagen I (1 : 400), collagen III (1 : 800), and 4HNE (1 : 200). The staining was analyzed using computerized morphometry (Image-Pro Plus 6.0 software, Media Cybernetics, Bethesda, MD, USA).

### 2.6. Real-Time Quantitative Polymerase Chain Reaction (PCR)

TRIzol (Invitrogen) was used to extract total RNA from the kidney tissues according to the manufacturer's instructions. A Revert Aid First Strand cDNA Synthesis Kit (Thermo Scientific, MA, USA) was used to reversely transcribe RNA into DNA. Real-time PCR was performed using a CFX96 Quantitative PCR Detection System. The primer sequences used for PCR amplification are summarized in Table 1.

### 2.7. Western Blotting

Protein was extracted from the kidney tissues or HK-2 cells, separated on 8–15% (according to the target protein) sodium dodecyl sulfate-polyacrylamide gel electrophoresis gels, and transferred onto polyvinylidene difluoride membranes (Millipore, Bedford, MA, USA). Antibodies against FN (1 : 1000), *α*-SMA (1 : 1000), E-cadherin (1 : 1000), collagen I (1 : 500), total OXPHOS complexes (1 : 400), SIRT1 (1 : 500), NOX4 (1 : 1000), SIRT3 (1 : 500), SOD2 (1 : 2000), and GAPDH (1 : 10000) were used for western blotting.

### 2.8. Transmission Electron Microscopy

After fixation in glutaraldehyde, the kidney tissues and HK-2 cells were postfixed in 1% osmium tetroxide, dehydrated in a graded alcohol series, and then embedded in Epon to prepare ultrathin sections (200–400 Å). The sections were stained with uranyl acetate and lead citrate and then examined using a digital electron microscope (JEM-1400; JEOL Ltd., Tokyo, Japan).

### 2.9. ATP Detection

ATP assay kits (Beyotime Biotechnology, Shanghai, China) were used to detect the ATP content in kidney tissues and HK-2 cells according to the manufacturer's instructions.

### 2.10. Detection of mtROS

HK-2 cells were washed twice with PBS and incubated for 30 min with 5 *μ*M mitoSOX Deep Red to detect mitochondrial ROS (mtROS). After removing the supernatant, the cells were washed twice with PBS and collected for centrifugation at 800 rpm for 5 min. The cells were then resuspended in 200 *μ*L PBS to analyze the average fluorescence intensity by flow cytometry. The data were obtained from three independent experiments.

### 2.11. Statistical Analysis

All data are expressed as means ± standard deviation. Comparisons between groups were analyzed by one-way ANOVA, and the comparisons between two groups were analyzed with a least significant difference test. *P* < 0.05 was considered statistically significant.

## 3. Results

### 3.1. AKF-PD-Derived Reduction of Renal Fibrosis in the UUO and IRI Models

**Figure 1:**
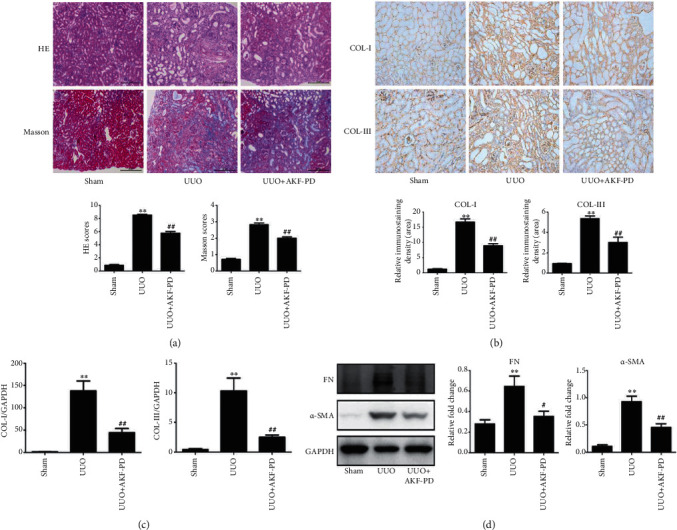
AKF-PD reduced renal fibrosis in the UUO model. (a) HE and Masson staining of renal tissues from the indicated mouse groups (*n* = 5); typical images (×200) for indicated groups are shown (upper panel). Quantifications for individual treatments (*n* = 5) are also provided (bottom panel). (b) Immunohistochemistry analyses of collagen I and collagen III in the indicated renal tissues. Typical images (×200) for individual groups and their quantifications (*n* = 4) are included. (c) Real-time PCR analyses for collagen I and collagen III expressions in the indicated mouse groups (*n* = 4). (d) The expression of FN and *α*-SMA in renal tissue from mice with the indicated treatments was measured by western blot. Typical images and quantification (*n* = 4) are shown. ^∗^*P* < 0.05 and ^∗∗^*P* < 0.01 UUO group vs. sham group; ^#^*P* < 0.05 and ^##^*P* < 0.01 UUO+AKF-PD group vs. UUO group. Data were analyzed by one-way ANOVA. AKF-PD: fluorofenidone; COL-I: collagen I; COL-III: collagen III; FN: fibronectin; *α*-SMA: alpha smooth muscle actin.

HE and Masson scores were used to evaluate tubulointerstitial injuries and interstitial ECM deposition separately [19]. HE and Masson scores were evidently increased in the UUO group, indicating tubulointerstitial injuries and interstitial ECM deposition, compared with the sham group (Figure 1(a)). In comparison to UUO mice treated with vehicle, mice treated with AKF-PD showed a partial but significant decrease in both renal tubular injury and interstitial ECM deposition (Figure 1(a)). UUO-induced ECM deposition was associated with elevations in collagen I and III depositions, revealed by immunohistochemistry and real-time PCR (Figures 1(b) and 1(c)); the depositions were significantly reduced by AKF-PD (Figures 1(b) and 1(c)). Additionally, upregulations of FN and *α*-SMA are typical events contributing to renal fibrosis [20]. In line with this knowledge, UUO led to increases in FN and *α*-SMA, which were significantly reduced by AKF-PD (Figure 1(d)).

**Figure 2:**
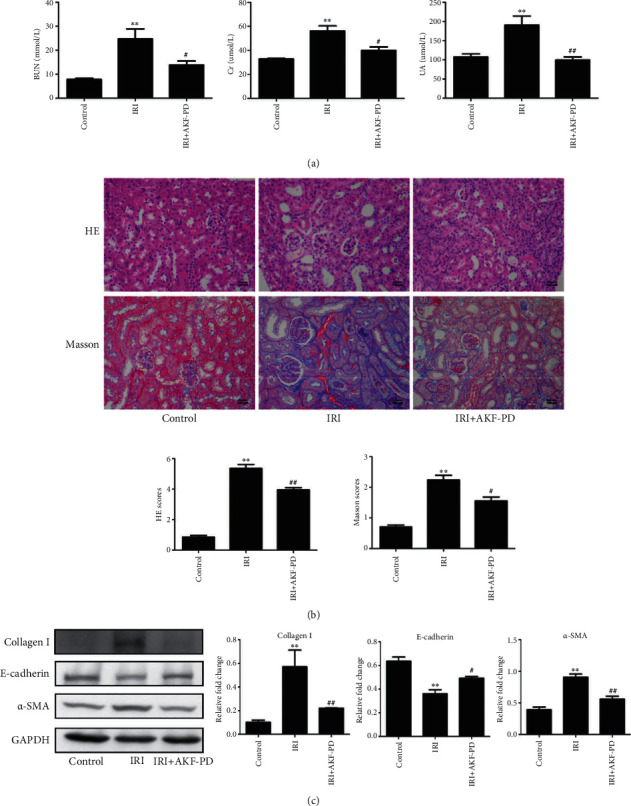
AKF-PD reduced renal fibrosis in the IRI model. (a) Serum urea nitrogen, creatinine, and uric acid from the indicated animal groups were determined and quantified (*n* = 5). (b) HE and Masson staining of renal tissues obtained from mice with the indicated treatments. Typical images (×200) and quantifications (*n* = 5) are shown. (c) The expression of collagen I, E-cadherin, and *α*-SMA in renal tissue from IRI mice was measured by western blot with typical images and quantifications (*n* = 4) included. ^∗^*P* < 0.05 and ^∗∗^*P* < 0.01 IRI group vs. control group; ^#^*P* < 0.05 and ^##^*P* < 0.01 IRI+AKF-PD group vs. IRI group. Data were analyzed by one-way ANOVA. AKF-PD: fluorofenidone; BUN: blood urea nitrogen; Cr: creatinine; UA: uric acid; *α*-SMA: alpha smooth muscle actin.

Furthermore, we also determined AKF-PD's effect on renal fibrosis in the IRI model. The ischemia insults delivered in this model resulted in significant increases in serum urea nitrogen, creatinine, and uric acid compared with the control group, revealing a decline in renal function (Figure 2(a)). Importantly, AKF-PD significantly preserved renal functions (Figure 2(a)). The IRI-caused decline in renal function was associated with tubulointerstitial injuries and interstitial ECM deposition (Figure 2(b)) along with upregulations of collagen I and with *α*-SMA and downregulation of E-cadherin (Figure 2(c)). All these events were reversed by AKF-PD (Figures 2(b) and 2(c)). Taken together, evidence reveals that AKF-PD reduced renal injuries at least in part via attenuation of renal fibrosis in the presence of urinary obstruction and IRI.

### 3.2. Reduction of Mitochondrial Damage by AKF-PD in the UUO and IRI Models

**Figure 3:**
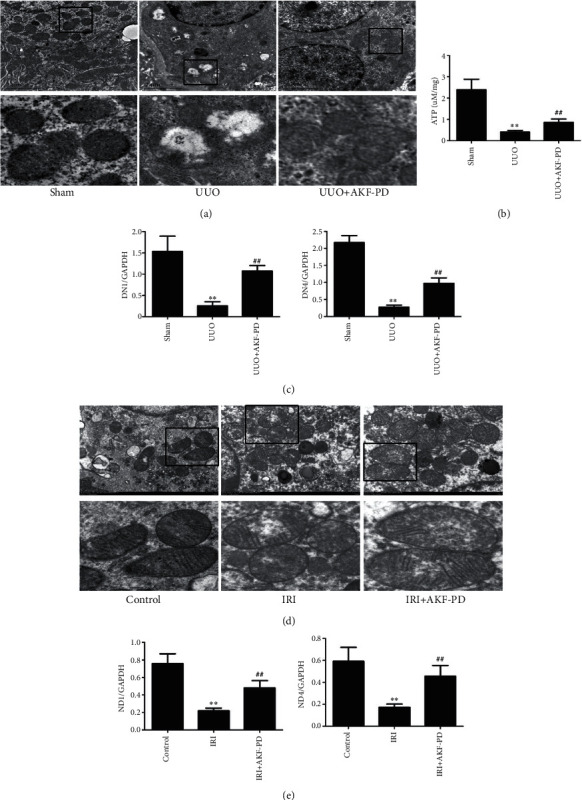
AKF-PD reduced mitochondrial damage in the UUO and IRI models. (a) Mitochondrial morphology of renal tubular epithelial cells from the indicated mouse group was examined by transmission electron microscopy. (b) ATP levels of renal tissue from the indicated mice were measured by the ATP assay kit and quantified (*n* = 4). (c) The expression of ND1 and ND4 in renal tissue from the indicated mice was measured by real-time PCR (*n* = 4). (d) Mitochondrial morphology of renal tubular epithelial cells from the indicated mouse group was examined by transmission electron microscopy. (e) The expression of ND1 and ND4 in renal tissue from the indicated mice was measured by real-time PCR (*n* = 4). ^∗^*P* < 0.05 and ^∗∗^*P* < 0.01 UUO/IRI group vs. sham/control group; ^#^*P* < 0.05 and ^##^*P* < 0.01 UUO+AKF-PD/IRI+AKF-PD group vs. UUO/IRI group. Data were analyzed by one-way ANOVA. AKF-PD: fluorofenidone; ND1: NADPH dehydrogenase subunit 1; ND4: NADPH dehydrogenase subunit 4.

In view of an important role of mitochondrial damage in renal fibrosis, renal protection of AKF-PD observed in both models above indicates a role of AKF-PD in protecting mitochondrial damage. We thus examined mitochondrial morphology using electron microscopy. In comparison to the sham group, mitochondria with degenerative changes including disruption in mitochondrial membrane and matrix swelling were observed in the UUO model, and these morphological manifestations were significantly improved by AKF-PD treatment (Figure 3(a)). Additionally, UUO treatment caused reductions in ATP production, as well as NADPH dehydrogenase subunit 1 (ND1) and NADPH dehydrogenase subunit 4 (ND4) (Figures 3(b) and 3(c)), which can be used to assess the mitochondrial DNA copy numbers [21]. These reductions were significantly attenuated by AKF-PD (Figures 3(b) and 3(c)). Our results showed AKF-PD can maintain mitochondrial morphology and increase ATP production and mitochondrial DNA copy numbers in UUO model mice.

The observed protection is not limited to UUO-induced mitochondrial damage. In the IRI model, we also observed disruption of mitochondrial membrane and matrix swelling (Figure 3(d)). AKF-PD maintained mitochondrial membrane integrity and ameliorated mitochondrial swelling (Figure 3(d)). Moreover, ND1 and ND4 were also reduced in the IRI group, compared with the control group, and AKF-PD uplifted the expression of ND1 and ND4 in the IRI model (Figure 3(e)). Evidence thus supports AKF-PD offers protection to damages of mitochondria caused by UUO and IRI.

### 3.3. AKF-PD Improving Mitochondrial Energy Metabolism in the UUO and IRI Models

**Figure 4:**
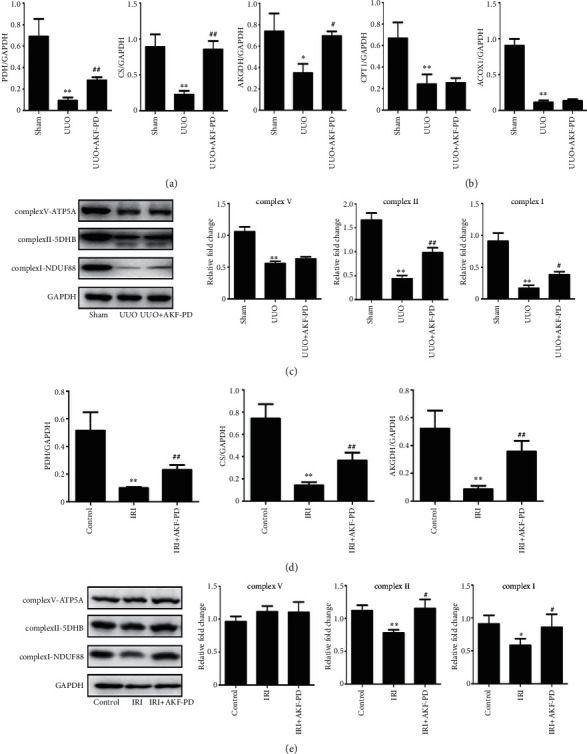
AKF-PD improved mitochondrial energy metabolism in the UUO and IRI models. (a) The expression of PDH, CS, and AKGDH in renal tissue from the indicated mice was measured by real-time PCR (*n* = 4). (b) The expression of CPT1 and ACOX1 in renal tissue from the indicated mice was determined by real-time PCR (*n* = 4). (c) The expression of complex I, complex II, and complex V in renal tissue from the indicated mice was analyzed by western blot (*n* = 4). (c) The expression of PDH, CS, and AKGDH in renal tissue from the indicated mice was examined by real-time PCR (*n* = 4). (d) The expression of complex I, complex II, and complex V in renal tissue from the indicated mice was measured by western blot (*n* = 4). ^∗^*P* < 0.05 and ^∗∗^*P* < 0.01 UUO/IRI group vs. sham/control group; ^#^*P* < 0.05 and ^##^*P* < 0.01 UUO+AKF-PD/IRI+AKF-PD group vs. UUO/IRI group. Data were analyzed by one-way ANOVA. AKF-PD: fluorofenidone; PDH: pyruvate dehydrogenase; CS: citrate synthase; AKGDH: *α*-ketoglutarate dehydrogenase; CPT1: carnitine palmitoyltransferase 1; ACOX1: acyl-CoA oxidase 1.

Mitochondria is the cellular structure to generate ATP through TCA cycle and FAO [22]. We suspected that AKF-PD might modulate these two metabolism pathways and thereby reserves ATP production in both UUO and IRI models. To examine this scenario, we first detected the key enzymes of TCA cycle, such as pyruvate dehydrogenase (PDH), citrate synthase (CS), and *α*-ketoglutarate dehydrogenase (AKGDH). Then, the key enzymes of FAO, including carnitine palmitoyltransferase 1 (CPT1) and acyl-CoA oxidase 1 (ACOX1), were also evaluated by real-time PCR. The expressions of these enzymes were decreased in the UUO model compared with the sham group (Figures 4(a) and 4(b)). AKF-PD significantly reduced the downregulation of PDH, CS, and AKGDH expression (Figure 4(a)) without affecting CPT1 and ACOX1 mRNA expressions (Figure 4(b)). Mitochondrial respiratory chain complexes regulate mitochondrial oxidative phosphorylation, which is also an important part of mitochondrial energy metabolism, like TCA cycle and FAO [22]. In comparison to the sham group, the expression of complex I, complex II, and complex V was decreased in the UUO model (Figure 4(c)). AKF-PD attenuated the reductions of complex I and complex II but not the downregulation of complex V (Figure 4(c)). Similar to the results observed in the UUO model, AKF-PD also reserved the expression of PDH, CS, AKGDH, complex I, and complex II in the IRI model and had no effect on the expression of complex V (Figures 4(d) and 4(e)).

### 3.4. AKF-PD Preserving Mitochondrial Biogenesis in the UUO and IRI Models

**Figure 5:**
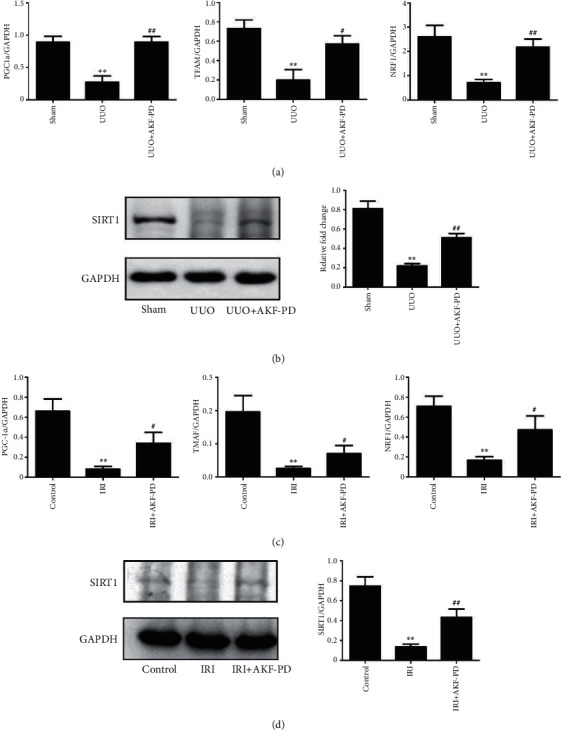
AKF-PD increased mitochondrial biogenesis in the UUO and IRI models. (a) The expression of PGC-1*α*, TFAM, and NRF1 in renal tissue from the indicated mice was measured by real-time PCR (*n* = 4). (b) The expression of SIRT1 in renal tissue from the indicated mice was measured by western blot (*n* = 4). (c) The expression of PGC-1*α*, TFAM, and NRF1 in renal tissue from the indicated mice was measured by real-time PCR (*n* = 4). (d) The expression of SIRT1 in renal tissue from the indicated mice was measured by western blot (*n* = 4). ^∗^*P* < 0.05 and ^∗∗^*P* < 0.01 UUO/IRI group vs. sham/control group; ^#^*P* < 0.05 and ^##^*P* < 0.01 UUO+AKF-PD/IRI+AKF-PD group vs. UUO/IRI group. Data were analyzed by one-way ANOVA. AKF-PD: fluorofenidone; PGC-1*α*: peroxisome proliferator-activated receptor *γ* coactivator-1*α*; TFAM: mitochondrial transcription factor A; NRF1: nuclear respiratory factor 1; SIRT1: sirtuin 1.

Next, we detected the effect of AKF-PD on mitochondrial biogenesis, which can regulate energy metabolism and offer protection to mitochondrial damage [23]. Compared with the sham group, the expression of peroxisome proliferator-activated receptor *γ* coactivator-1*α* (PGC-1*α*), SIRT1, and their downstream transcription, such as factors mitochondrial transcription factor A (TFAM) and nuclear respiratory factor 1 (NRF1), was decreased in the UUO group, and these changes were significantly reversed by AKF-PD (Figures 5(a) and 5(b)). The expressions of the above mitochondrial biogenesis factors were also decreased in the IRI model compared with the control group, and AKF-PD evidently preserved the expression of these mitochondrial biogenesis factors in the IRI model (Figures 5(c) and 5(d)).

### 3.5. AKF-PD-Mediated Suppression of Mitochondrial Oxidative Stress in the UUO and IRI Models

**Figure 6:**
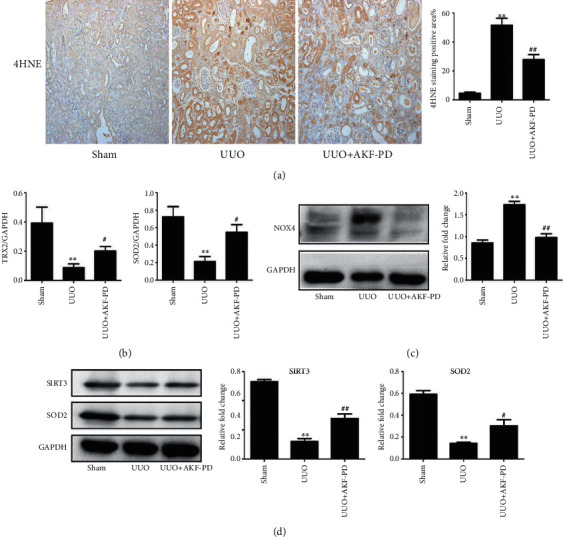
AKF-PD suppressed mitochondrial oxidative stress in the UUO model. (a) The expression of 4HNE in renal tissue from the indicated mice was determined by immunohistochemistry, ×200 (*n* = 4). (b) The expression of TRX2 and SOD2 in renal tissue from the indicated mouse group was measured by real-time PCR (*n* = 4). (c, d) The expression of NOX4, SIRT3, and SOD2 in renal tissue from the indicated mice was measured by western blot (*n* = 4). ^∗^*P* < 0.05 and ^∗∗^*P* < 0.01 UUO group vs. sham group; ^#^*P* < 0.05 and ^##^*P* < 0.01 UUO+AKF-PD group vs. UUO group. Data were analyzed by one-way ANOVA. AKF-PD: fluorofenidone; 4HNE: 4-hydroxynonenal; TRX2: thioredoxin 2; SOD2: superoxide dismutase 2; NOX4: NADPH oxidase 4; SIRT3: sirtuin 3.

**Figure 7:**
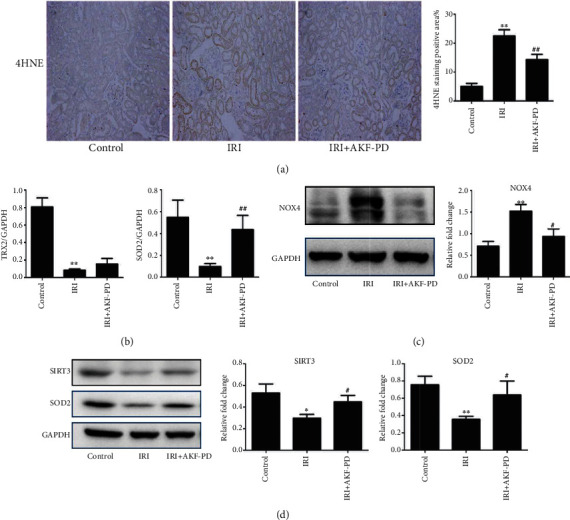
AKF-PD suppressed mitochondrial oxidative stress in the IRI model. (a) The expression of 4HNE in renal tissue from the indicated mice was determined by immunohistochemistry, ×200 (*n* = 4). (b) The expression of TRX2 and SOD2 in renal tissue from the indicated mouse group was measured by real-time PCR (*n* = 4). (c, d) The expression of NOX4, SIRT3, and SOD2 in renal tissue from the indicated mice was measured by western blot (*n* = 4). ^∗^*P* < 0.05 and ^∗∗^*P* < 0.01 IRI group vs. control group; ^#^*P* < 0.05 and ^##^*P* < 0.01 IRI+AKF-PD group vs. IRI group. Data were analyzed by one-way ANOVA. AKF-PD: fluorofenidone; 4HNE: 4-hydroxynonenal; TRX2: thioredoxin 2; SOD2: superoxide dismutase 2; NOX4: NADPH oxidase 4; SIRT3: sirtuin 3.

Mitochondria are the main source of oxygen free radicals, which facilitate oxidative stress occurrence in cells [24]. Suppressing of mitochondrial oxidative stress sets to reduce mitochondrial damage and sustain ATP production in the UUO and IRI models [25, 26]. In this regard, upregulation of 4HNE (a product of lipid peroxidation) occurred in the UUO model compared with the sham group, and AKF-PD significantly decreased the expression of 4HNE (Figure 6(a)). Then, we explored the effect of AKF-PD on mitochondrial redox-related proteins in the UUO model. In comparison to the sham group, the expression of thioredoxin 2 (TRX2), SOD2, and SIRT3 was reduced, whereas NOX4 expression was increased in the UUO model. AKF-PD partly reversed these changes (Figures 6(b)–6(d)). Additionally, AKF-PD had a similar effect in the IRI model, where it decreased the expression of 4HNE and NOX4 and increased the expression of SIRT3 and SOD2, but had no effect on the expression of TRX2 (Figures 7(a)–7(d)).

.

### 3.6. AKF-PD Reducing Mitochondrial Damage and Mitochondrial Oxidative Stress in HK-2 Cells

**Figure 8:**
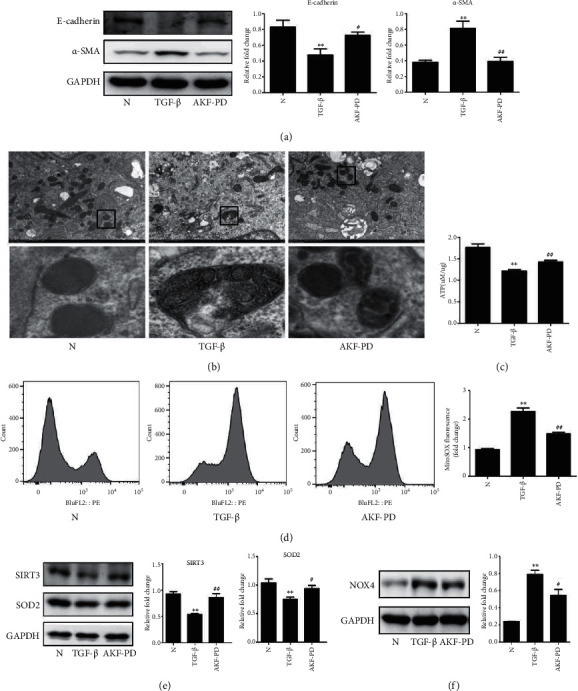
AKF-PD reduced mitochondrial damage and mitochondrial oxidative stress in HK-2 cells. (a) The expression of E-cadherin and *α*-SMA in HK-2 cells was measured by western blot (*n* = 3). (b) Mitochondrial morphology of HK-2 cells was examined by transmission electron microscopy. (c) ATP levels of HK-2 cells were measured by the ATP assay kit (*n* = 3). (d) MitoSOX Deep Red fluorescence was used to detect mitochondrial ROS of HK-2 cells by flow cytometry analysis (*n* = 3). (e, f) The expression of SIRT3, SOD2, and NOX4 in HK-2 cells was measured by western blot (*n* = 3). ^∗^*P* < 0.05 and ^∗∗^*P* < 0.01 TGF-*β* group vs. normal group; ^#^*P* < 0.05 and ^##^*P* < 0.01 AKF-PD group vs. TGF-*β* group. Data were analyzed by one-way ANOVA. AKF-PD: fluorofenidone; N: normal group; TGF-*β*: transforming growth factor-*β*; *α*-SMA: alpha smooth muscle actin; SOD2: superoxide dismutase 2; NOX4: NADPH oxidase 4; SIRT3: sirtuin 3.

To further investigate the protective effect of AKF-PD on mitochondrial damage in renal tubular epithelial cells, we used TGF-*β* to stimulate HK-2 cells, human kidney epithelial cells. The results showed that TGF-*β* stimulation decreased the expression of E-cadherin and increased the expression of *α*-SMA in HK-2 cells, which suggested that TGF-*β* induced EMT (Figure 8(a)). AKF-PD significantly increased the expression of E-cadherin and decreased the expression of *α*-SMA, resisting EMT occurrence (Figure 8(a)). The electron microscopy results showed that disrupted mitochondrial membrane and matrix swelling existed in TGF-*β*-stimulated HK-2 cells, and AKF-PD significantly improved these morphological manifestations (Figure 8(b)). Furthermore, AKF-PD increased ATP contents in HK-2 cells (Figure 8(c)). Additionally, compared to the normal group, stimulation with TGF-*β* induced the production of excessive amounts of mtROS in HK-2 cells, as revealed by the fluorescence of mitoSOX Red, while treatment with AKF-PD significantly inhibited the production of mtROS (Figure 8(d)). TGF-*β* stimulation also decreased the expression of SIRT3 and SOD2 and increased the expression of NOX4 in HK-2 cells, and AKF-PD partly reversed these changes (Figures 8(e) and 8(f)). Evidence supports a role of AKF-PD in reducing mitochondrial oxidative stress of HK-2 cells treated with TGF-*β*.

## 4. Discussion

Renal fibrosis is a common pathological feature of progressive CKD [27], and clinical management of renal fibrosis remains challenging, which is in part attributed to the lack of effective drugs. Accumulative evidence reveals an important role of mitochondrial damage in renal fibrosis, such as IRI and urinary obstruction [6, 7]. AKF-PD displays antirenal fibrosis activities [28, 29]. This renal protection might be in part owing to AKF-PD actions in protecting mitochondrial damage; this possibility is consistent with our previous research suggesting a role of AKF-PD in reducing mitochondrial damage in a folic acid-induced renal fibrosis model [16]. In this study, we have extended this theme in the setting of renal fibrosis caused by UUO and IRI. By attenuating renal pathological changes and reducing the expressions of fibrosis-related proteins, including collagen I, collagen III, FN, and *α*-SMA, we can confirm that AKF-PD has an antifibrotic effect in UUO and IRI models. Specifically, AKF-PD sustains mitochondrial integrity and functions in renal tissues under two typical fibrotic insults, UUO and IRI.

Mitochondria play essential roles in kidney health. In the kidney, fatty acids are important substrates of ATP production [30] that are converted to acyl-CoA by acyl-CoA synthetases and then transported to mitochondria by FAO enzymes, such as CPT-1 and ACOX1. In the TCA cycle, pyruvate from glycolysis is converted into acetyl-CoA by PDH, and then, acetyl-CoA is metabolized to carbon dioxide by enzymes such as CS and AKGDH [31]. The electrons generated in the TCA cycle undergo oxidative phosphorylation to produce ATP [31]. Our results showed that TCA cycle and mitochondrial respiratory chain may play a role in progression of renal fibrosis induced by UUO and IRI. Importantly, AKF-PD preserved the expression of PDH, CS, and AKGDH along with the expression of mitochondrial respiratory chain complexes (complex I and complex II) in the UUO and IRI models. In view of AKF-PD having no effect on the key enzymes of FAO in the UUO model, we would favor the scenario that AKF-PD affects some signaling pathways involved in TCA cycle and mitochondrial respiratory chain. This may explain that AKF-PD offers partial protection of mitochondria from damage in the UUO and IRI models.

In addition to mitochondrial metabolism, mitochondrial biogenesis and mitochondrial oxidative stress are also critical in renal fibrosis. Mitochondrial biogenesis can be activated by energy demand and is intimately connected to mitochondrial energy metabolism [32]. The SIRT1/PGC-1*α* signaling pathway, one of the core mechanisms of mitochondrial biogenesis [33, 34], can enhance respiratory chain complex expression and increase mitochondrial DNA copy numbers via facilitating NRF1 and TFAM expression [35, 36]. In this research, AKF-PD reserves the expression of SIRT1, PGC-1*α*, TFAM, and NRF1 in the UUO and IRI models, which at least partly explained its ability in increasing respiratory chain complex expression and mitochondrial DNA copy numbers. Oxidative stress is not only a result of mitochondrial damage but also a cause of mitochondrial damage. The damage is attributed to massive mtROS that disturbs mitochondrial structure and inhibits mitochondrial function, causing abnormal mitochondrial morphology and impaired ATP production [37]. Furthermore, mitochondrial oxidative damage promotes ECM deposition in the UUO and IRI models [38, 39]. 4HNE is a common product of lipid peroxidation and a surrogate marker of oxidative stress in tissues [40, 41]. TRX2 and NOX4 as well as the SIRT3/SOD2 signaling pathway play roles in regulating mtROS production in mitochondria [42, 43]. The role of NOX4, but not TRX2 and SIRT3/SOD2 signaling pathway, in UUO and IRI-induced nephropathy has been reported [44, 45]. This study provides the first evidence that inhibition of TRX2 expression and SIRT3/SOD2 signaling pathway contributed to mitochondrial damage in the UUO and IRI models. Furthermore, AKF-PD can reduce the expression of 4HNE and NOX4, increase the expression of SIRT3 and SOD2, and have no evident increasing effect on TRX2 in the UUO and IRI models. Collectively, our research supports AKF-PD in sustaining mitochondrial biogenesis and preventing oxidative stress as a part of AKF-PD actions in protecting mitochondrial damage. AKF-PD's mitochondria protection is strengthened by its activity in maintaining mitochondrial functions in human kidney tubular epithelial HK-2 cells treated with TGF-*β*, an established profibrotic factor.

Although mitochondrial damage has been reported in the UUO and IRI models [6, 7], the characteristics of mitochondrial damage in the two renal fibrosis models remained vague because of the complexity of the injuring mechanism. Our results suggested that impaired TCA cycle and mitochondrial respiratory chain as well as inhibited TRX2 expression and SIRT3/SOD2 signaling pathway contributed to mitochondrial damage in the UUO and IRI models. Furthermore, we for the first time confirmed that AKF-PD inhibited IRI-induced renal fibrosis. What is more, we provide a comprehensive set of evidence supporting AKF-PD's actions in sustaining mitochondrial integrity and function, through which AKF-PD displays antifibrotic activities in different renal disease settings. This research supports AKF-PD's clinical applications in managing patients with renal fibrosis.

## 5. Conclusion

In summary, we found that AKF-PD inhibited renal fibrosis at least in part via reducing mitochondrial damage in the UUO and IRI models. The underlying mechanism was related to (1) sustaining mitochondrial energy metabolism by elevating of TCA cycle enzymes and mitochondrial respiratory chain complexes, (2) improving mitochondrial biogenesis with activation of the SIRT1/PGC-1*α* signaling pathway, and (3) reducing mitochondrial oxidative stress likely via enhancing the SIRT3/SOD2 signaling pathway and reducing the expression of NOX4. This study provides new theoretical and experimental evidence for AKF-PD as a therapeutic candidate for renal fibrosis.

## Figures and Tables

**Table 1:** RT-PCR primer sequences.

Name	Forward (5′ to 3′)	Reverse (5′ to 3′)
Mouse		
Collagen I	GTCCCAACCCCCAAAGAC	CATCTTCTGAGTTTGGTGATACGT
Collagen III	GAAGTCTCTGAAGCTGATGGG	TTGCCTTGCGTGTTTGATATTC
GAPDH	TGACCTCAACTACATGGTCTACA	CTTCCCATTCTCGGCCTTG
ND1	CACCCCCTTATCAACCTCAA	ATTTGTTTCTGCGAGGGTTG
ND4	ATTATTATTACCCGATGAGGGAACC	ATTAAGATGAGGGCAATTAGCAGT
PDH	GAAATGTGACCTTCATCGGCT	TGATCCGCCTTTAGCTCCATC
CS	GGGACTTGTGTATGAGACTTCG	AGCCAAAATAAGCCCTCAGG
AKGDH	GTTTCTTCAAACGTGGGGTTCT	GCATGATTCCAGGGGTCTCAAA
CPT1	GGTCTTCTCGGGTCGAAAGC	TCCTCCCACCAGTCACTCAC
ACOX1	CTTGGATGGTAGTCCGGAGA	TGGCTTCGAGTGAGGAAGTT
TFAM	GGAATGTGGAGCGTGCTAAAA	TGCTGGAAAAACACTTCGGAATA
NRF1	CGCAGCACCTTTGGAGAA	CCCGACCTGTGGAATACTTG
PGC-1*α*	TATGGAGTGACATAGAGTGTGCT	CCACTTCAATCCACCCAGAAAG
TRX2	GCTAGAGAAGATGGTCGCCAAGCAGCA	TCCTCGTCCTTGATCCCCACAAACTTG
SOD2	GTGTCTGTGGGAGTCCAAGG	CCCCAGTCATAGTGCTGCAA

## Data Availability

The data used to support the findings of this study are available from the first author and corresponding author upon reasonable request.
